# Regular exercise augments gut-brain communication and visceral feedback control of appetite

**DOI:** 10.1016/j.molmet.2026.102463

**Published:** 2026-10-28

**Authors:** Diba Borgmann, Dylan M. Rausch, Sofia Lundh, Shad Hassan, Cansu Tokgöz, Annika Glatzel, Leonie Cabot, Malou C. ten Voorde, Cecilie Bæch-Laursen, Paula Sanchis Tortosa, Claus Brandt, Heiko Backes, Tune H. Pers, Henning Fenselau

**Affiliations:** 1 Novo Nordisk Foundation Center for Basic Metabolic Research, Central Regulation of Metabolism, Faculty for Health and Medical Sciences, University of Copenhagen, Denmark; 2 Max Planck Institute for Metabolism Research, Synaptic Transmission in Energy Homeostasis, Cologne, Germany; 3 Center for Physical Activity Research, Rigshospitalet, University of Copenhagen, Denmark; 4 Max Planck Institute for Neurobiology of Behavior, Behavior and Brain Organization, Bonn, Germany; 5 Novo Nordisk A/S, Copenhagen, Denmark; 6 Max Planck Institute for Metabolism Research, Multimodal Imaging, Cologne, Germany; 7 Excellence Cluster on Cellular Stress Responses in Aging Associated Diseases (CECAD), University of Cologne, Joseph-Stelzmann-Str. 26, 50931 Cologne, Germany; 8 Policlinic for Endocrinology, Diabetes, and Preventive Medicine, University Hospital Cologne, Kerpener Str. 26, 50924 Cologne, Germany

**Keywords:** Exercise, Gut-brain axis, Feeding behavior, Brainstem, FIBCD1, Energy expenditure

## Abstract

**Objectives:**

Regular exercise is widely accepted to support long-term body weight management; however, the neurobiological adaptations that contribute to the beneficial effects of habitual physical activity beyond increases in energy expenditure per se remain unclear. Here, we investigated whether regular exercise alters gut-brain communication and visceral feedback control of feeding.

**Methods:**

Mice were subjected to regular voluntary wheel running. Energy expenditure and feeding were assessed under basal conditions and following an overnight fast. Nutrients were delivered directly into the stomach by intragastric infusion. Brain-wide responses to intragastric nutrient delivery were assessed using positron emission tomography (PET). Single-cell sequencing and histological analyses were used to determine nutrient-responsive neuronal populations in the caudal brainstem. Genetic associations with body weight were evaluated using GWAS data.

**Results:**

Intragastric nutrient delivery suppressed feeding more strongly in exercising mice. Brain-wide PET revealed enhanced responses to intragastric nutrients in various brain regions. Single-cell sequencing identified several nutrient-responsive caudal brainstem populations, including a previously unrecognized population marked by expression of fibrinogen C domain containing 1 (FIBCD1). Regular exercise selectively enhanced the nutrient-evoked activation of FIBCD1 neurons. Histological analyses demonstrate that FIBCD1 neurons are also present in the human brainstem, and GWAS data suggest a potential relevance of this neuron population to body-weight regulation.

**Conclusions:**

Regular exercise enhances behavioral and brain responses to intragastric nutrient delivery and sensitizes FIBCD1 neurons in the brainstem of mice. These findings reveal an exercise-associated adaptation of gut-brain communication that may contribute to improved matching of caloric intake to energy expenditure.

## Highlights

• Regular exercise enhances visceral feedback control of appetite.
• Exercise augments brain responses to gut-derived nutrient signals.
• FIBCD1 neurons are a novel gut nutrient-responsive brainstem population.
• Exercise selectively sensitizes FIBCD1 neurons to gut nutrient signals.
• FIBCD1 marks a human brainstem population linked to body weight regulation.

## 1 Introduction

Overconsumption of calories paired with a sedentary lifestyle is the main driver of obesity. Regular exercise is a well-established recommendation for supporting long-term body-weight control [1], [2], [3]. One of the primary mechanisms through which exercise facilitates weight management is by increasing energy expenditure (EE) [4]. In humans, structured physical activity elevates total daily EE, with moderate-intensity exercise contributing ∼500 kcal per session on average, depending on factors such as duration, intensity, and individual metabolic differences [5]. Despite the resulting caloric deficit, lean individuals who engage in regular exercise often do not exhibit a proportional compensatory rise in energy intake [6], [7]. As a result, regular exercisers often maintain a stable or negative energy balance over time. This phenomenon is observed even in obesogenic environments, where access to energy-dense foods is abundant. Furthermore, exercise as an add on therapy to pharmacological weight loss interventions results in augmented body-weight reduction, and decreased weight regain after cessation of medication [8]. Findings from experimental models support effects of regular exercise beyond EE [9], [10]. Rodents subjected to regular physical activity, such as voluntary wheel running, are more likely to maintain a stable body weight compared to sedentary counterparts, even when provided a high-fat diet, indicating the role of exercise in mitigating diet-induced body-weight gain [9], [10]. Together, these observations suggest that regular exercise may engage physiological and behavioral adaptations that influence energy balance beyond increasing EE. Understanding these mechanisms may help optimize exercise-based interventions for obesity prevention and treatment.

However, studies examining the effects of exercise on appetite and body-weight regulation in humans have yielded heterogeneous results. While some studies report improved appetite regulation and reduced caloric compensation in habitual exercisers, others found partial or complete compensation of exercise-induced EE through increased food intake [11], [12], [13], [14]. Therefore, self-reported dietary assessments, such as questionnaires, may not capture the intrinsic physiological adaptations that link exercise to feeding behavior. Nevertheless, more controlled experimental approaches, such as preload paradigms, have provided valuable insights [15], [16]. These studies assessed compensatory changes in food intake by measuring how individuals adjust their subsequent consumption after a fixed-calorie food item. In this context, previous studies found that habitual exercisers consume less food following a high-caloric preload compared to sedentary individuals when blinded to caloric values [15], [16]. These findings suggest that regular exercise enhances the ability to integrate additionally consumed calories into subsequent eating behavior. The neurobiological mechanism underlying this adapted feeding regulation remains unclear.

A key system involved in food intake regulation is the gut-brain axis [17], [18], [19], [20], [21], [22], [23], [24], [25]. Among other things, this axis serves to transmit signals from gastrointestinal organs to the brain to regulate, in turn, further consumption [25]. As such, it is a candidate communication pathway whose modulation by regular exercise could contribute to adaptations in feeding behavior. During a meal, the gut-brain axis relays mechanical information about stretch and distension of gastrointestinal organs, thereby providing an early estimate about the volume of ingested food [24], [26], [27]. In addition, sensing and transmission of chemosensory signals convey information about nutrient availability in the gut upon food consumption [19], [23], [26], [28]. Two primary mediators of this visceral feedback signaling are gut peptides that are secreted from enteroendocrine cells [29], [30], [31], and sensory neurons of the vagus nerve, which innervate the stomach and intestines with their peripheral endings [17], [32], [33], [34]. The dorsal vagal complex, which contains the area postrema (AP) and nucleus of the solitary tract (NTS), is a primary relay site for nutrient signals mediated via the gut-brain axis. AP and NTS neurons are activated upon mechanical stimulation along the GI tract or intestinal nutrient detection, and manipulation studies have demonstrated the necessity of these cells in the visceral feedback regulation of feeding [24], [26], [27], [35], [36], [37]. This gut-to-brain communication is also crucial to promote termination of ongoing meals, thereby restricting the rate at which nutrients reach the gut to promote efficient nutrient digestion and absorption.

Whether and how gut-brain communication is modulated by regular exercise is unclear, in part due to technical challenges associated with isolating cues that emerge in gastrointestinal organs. To overcome these difficulties, we employed an intragastric infusion protocol for the delivery of nutrients directly into the stomach of mice, thereby bypassing sensory cues, such as smell and taste associated with food consumption. We found that mice subjected to regular voluntary wheel running show an enhanced reduction in food intake following intragastric nutrient delivery. Moreover, using activity measurements we demonstrate that gut nutrient-evoked brain responses in various regions associated with visceral feedback control of appetite are augmented in regular runners. Our results indicate that regular exercise is associated with enhanced neural and behavioral responses to gut nutrient stimuli, and support the possibility that exercise alters gut-brain communication involved in feeding regulation.

## 2 Results

### 2.1 Regular voluntary wheel running increases energy expenditure without full caloric compensation

To study feeding adaptations to regular physical activity, we employed a voluntary wheel running protocol in mice. Age- and body-weight-matched mice were given unrestricted access to a running wheel or a sham wheel (control) in their home cages, followed by analyses of feeding, body-weight and -composition (Figure 1A). After 4 weeks, running mice showed lower body weight, which was driven by a reduction in body fat, but not lean mass (Figure 1B–D, S1A, B). Body weight was reduced in the first week of voluntary wheel running, while weight gain over the following weeks occurred parallel (Figure 1E). Thus, consistent with previous observations [9], [10], [38], voluntary wheel running results in a negative energy balance in mice.

**Figure 1:**
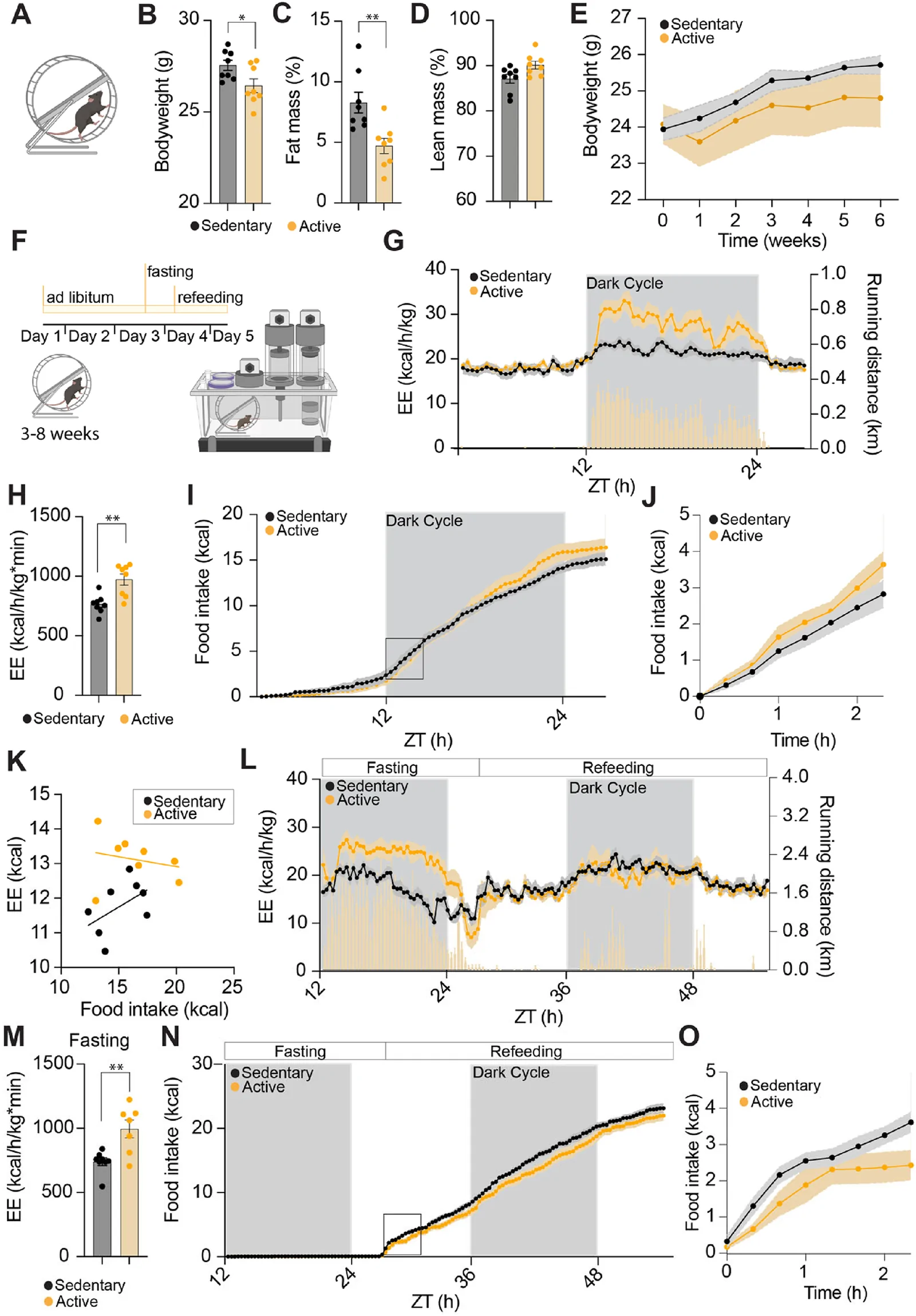
**Increased energy expenditure in regularly running mice is not compensated by increased food intake.A,** Age- and body-weight matched mice were subjected to a voluntary wheel running (active) or a sham (sedentary) protocol for 4 weeks, followed by body-weight and -composition measurements. **B,** After 4 weeks, active mice showed significantly lower body weights compared to sedentary littermates. ∗p = 0.0326 (unpaired two-sided t-test). n = 8 mice. **C, D,** Fat **(C)**, but not lean mass **(D)** is decreased in active mice after 4 weeks of voluntary wheel running. ∗∗p = 0.0044 (unpaired two-sided t-test). n = 8 mice. **E,** Body-weight development over 6 weeks of voluntary wheel running (active) and sedentary controls. After an initial drop-rebound of body weights during weeks 1–3, active mice are about 1g below the control group. n = 5–7 mice. **F,** Schematic of the experimental approach to measure EE, food intake, and gross locomotor activity in mice subjected to regular, voluntary wheel running. Control mice (sedentary) had access to a non-moveable wheel. After 3–8 weeks of baseline, phenotypic parameters were recorded in custom-made metabolic cages during ad libitum food access (Day 1–2), during a 16 h overnight fast (Day 3), and during food intake after fasting (refeeding; Day 4–5). **G,** With ad libitum food access, mice with running wheels (active), increased running during the dark cycle (bar graphs, right y-axis), resulting in an increase in EE. n = 8–9 mice. **H,** Area under the curve (AUC) for EE (kcal/h/kg) normalized for body weight during the dark cycle is increased in active mice. ∗∗∗p = 0.0068 (unpaired two-sided t-test). n = 8–9 mice. **I,** Food intake during ad libitum conditions. Active mice moderately increase food intake in the second half of the dark cycle, which did not reach significance. p = 0.6523 (two-way ANOVA). n = 8–9 mice. **J,** Cumulative food Intake normalized to the beginning of the dark cycle shows a moderate, not significant increase in cumulative food intake over the first 2 h of the dark cycle. p = 0.2569 (two-way ANOVA). n = 8–9 mice. **K,** Correlation of 24-hour EE and food intake for active and sedentary mice. Despite wheel running-induced increases in EE, physically active mice did not compensate for the caloric deficit. n = 8–9 mice. **L,** During fasting, mice with running wheels (active) increased running during the dark cycle (bar graphs, right y-axis), resulting in an increase in EE. n = 7–8 mice. **M,** AUC for EE normalized for body weight during fasting of active mice is significantly increased. ∗∗p = 0.0038 (two-sided unpaired t-test). n = 7–8 mice.**N,** Food intake during refeeding following a 16 h overnight fast. Active mice did not compensate the caloric deficit via an increase in energy intake over 24 h of refeeding. n = 7–8 mice. **O,** Active mice show a moderate reduction in feeding behavior during the first 2 h of refeeding after a 16-h overnight fast. p = 0.2804 (two-way ANOVA). n = 7–8 mice. Values are presented as mean ± SEM. Circles represent individual animals. Mice are from multiple litters.

Next, we analyzed wheel running activity, EE, overall physical activity, and feeding behavior in mice placed in metabolic cages (TSE Phenomaster) after 3–8 weeks of regular wheel running (Figure 1F). We found that during the light cycle, mice did not engage in wheel running, and that EE was not different when compared with controls (Figure 1G). At the beginning of the dark cycle, wheel running activity rapidly increased and remained elevated until the beginning of the next light cycle, causing an overall increase of EE during the dark cycle (Figure 1G,H). This corresponded to an EE increase of 1.35 kcal over 24 h in active mice (Fig. S1C). We did not observe changes in respiratory exchange ratio (RER; Fig. S1D), indicating no differences in fuel type selection between runners and sedentary controls.

Food intake analysis revealed that 24 h food consumption was not significantly different between groups (Figure 1I, S1E); yet, we observed that physically active mice showed a slight increase in dark cycle feeding during the first 2 h when normalized to the onset of the dark cycle (Figure 1J). Regular runners also showed a tendency of larger 20-minute feeding bouts, which did not reach statistical significance (Fig. S1F). The number of calories consumed over 24 h was not correlated to calories expended over this time, although comparison between experimental groups revealed opposite trends, with high performing runners showing the lowest caloric intake (Figure 1K). Thus, regular running mice do not show a proportional increase in food intake relative to elevated EE.

We next subjected mice to a fasting-refeeding paradigm to assess whether elevated post-fast refeeding upon the reintroduction of food is altered by regular exercise (Figure 1F). Running activity increased during the dark phase of the fasting period, which resulted in a significant increase in EE, corresponding to about 2 kcal (sedentary = 6.39 kcal, active = 8.16 kcal, on average) compared to sedentary controls (Figure 1L, M, S1G). Consistent with this increased EE in absence of food, body-weight loss during the overnight fast was larger in mice with running wheels compared to sedentary controls (Fig. S1H). Running activity during fasting was significantly higher compared to ad libitum conditions (Fig. S1I).

Feeding analysis showed that 24-hour food intake upon reintroduction of food was not different between groups (Figure 1N). In addition, we observed that food intake during the first 2 h after food became available was slightly decreased in wheel running mice (Figure 1O), corroborated by a decrease in 20-minute feeding bout size, which was significant in the first 20 min of refeeding (Fig. S1J). In both groups acute food intake during the first 2 h of refeeding did not correlate with fasting EE (Fig. S1L), and active mice showed a more modest body-weight recovery (Fig. S1H) with lower levels of overcompensation after 24 h of refeeding (Fig S1L). Running activity on the day following the fasting period was reduced (Figure 1L). Wheel running mice also showed a decrease in respiratory exchange ratio (RER) in the day following the fasting period (Fig. S1M, N), suggesting increased fatty acid oxidation during energy repletion. During ad libitum conditions as well as fasting, EE strongly correlated with wheel running activity (Fig. S1O).

Together, these findings demonstrate that wheel running mice do not increase post-fast-food intake when challenged by an overnight fasting paradigm, despite elevated EE. Instead, we observed an acute reduction in feeding when food became available after fasting.

### 2.2 Feeding behavior and brain responses upon gut nutrient delivery

The acute reduction in post-fast feeding in regular runners could be attributed to enhanced gut-to-brain transmission of nutrient signals, which can rapidly reduce appetite [24], [33], [39]. To explore this possibility, we equipped mice with an intragastric catheter for the infusion of a nutrient solution (Figure 2A) - an approach that enables administration of a precise amount of calories and nutrients directly into the stomach [40], [41]. We determined food intake after an overnight fast following intragastric infusion of 0.5 ml (0.5 kcal) of the liquid diet Ensure (1 kcal/mL, flow rate 6 mL/h; 45% energy from carbohydrates, 20% from protein, 35% from fat; Figure 2B). Intragastric infusion of Ensure 30 min prior to providing access to food reduced post-fast refeeding in regularly running mice (Figure 2B,C). The reduction in feeding was particularly apparent at the first measurement after 30 min, where regular runners showed a marked reduction in food intake (Figure 2C). While sedentary mice also reduced food intake, this did not reach significance, and analysis of the consumed calories after 2 h showed that feeding reduction in controls matched the 0.5 kcal that was provided by the intragastrically delivered Ensure solution (Figure 2C). Thus, intragastric nutrient delivery more potently reduces post-fast refeeding in regularly exercising mice (Figure 1O, S1J).

**Figure 2:**
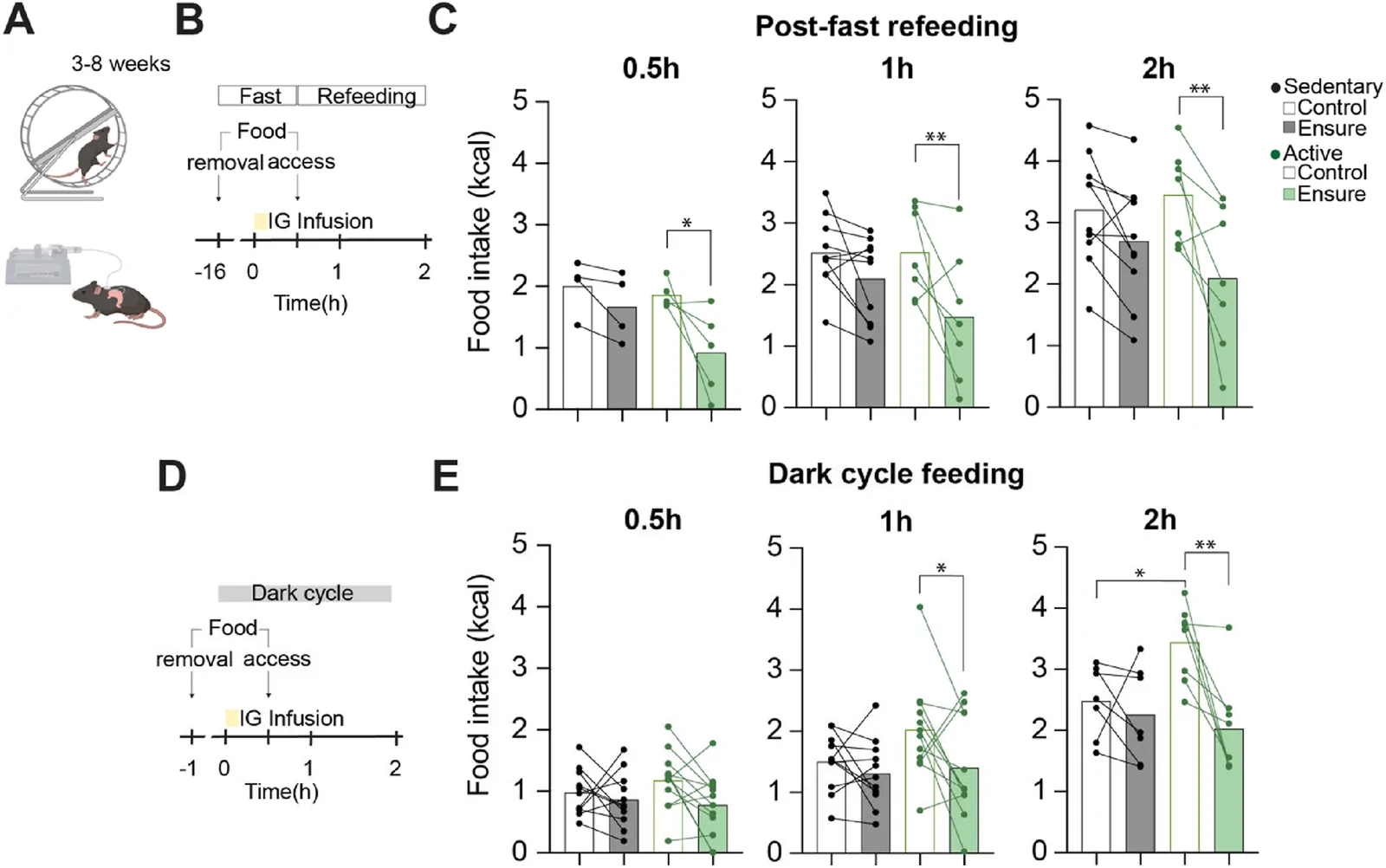
**Regular exercise augments feeding suppression to intragastric nutrient delivery.A,** Protocol schematic. Mice were subjected to a voluntary wheel running (active) or sham (sedentary) protocol. A gastric catheter was implanted for the delivery of Ensure. Food intake measurements were performed after 3–8 weeks of running. **B,** Schematic of the experimental design. 16 h overnight fasted mice received an intragastric delivery of 0.5 ml (0.5 kcal) of the liquid diet Ensure (yellow bar) 30 min prior to reintroducing food. **C,** Cumulative food intake in fasted mice 0.5 h, 1 h, and 2 h after reintroducing food (Post-fast refeeding) following intragastric Ensure delivery. 0.5 h: ∗p = 0.0330 1 h: ∗∗p = 0.0051; 2 h: ∗∗p = 0.0013 (two-way ANOVA with Sidak post hoc test). n = 4–9 mice. **D,** Schematic of the experimental design. Mice received an intragastric delivery of 0.5 ml (0.5 kcal) of the liquid diet Ensure (yellow bar) at the beginning of the dark cycle (grey bar). Food was removed 1 h before, and reintroduced 30 min after the start of infusion. **E,** Cumulative food intake in sedentary and active mice after 0.5 h, 1 h and 2 h of food access following intragastric Ensure administration at the beginning of the dark cycle. 1 h: ∗p = 0.0378; 2 h: ∗p = 0.0255, ∗∗p = 0.0022 (two-way ANOVA with Sidak post hoc test). n = 7–8 mice. Values are presented as mean ± SEM. Circles represent individual animals. Mice are from multiple litters.

We next measured feeding following intragastric nutrient infusion at the onset of the dark cycle (Figure 2D), a time period where regular runners showed numerically higher baseline food intake (Figure 1J), likely reflecting the elevated energetic demands associated with voluntary running during the active phase. In sedentary controls, we found that intragastric infusion did not alter food intake during the first 2 h of the dark cycle (Figure 2E). By contrast, food intake in regular wheel running mice was reduced, which reached significance at the 1- and 2-hour time points (Figure 2E). Overall comparison of food intake between active and sedentary mice demonstrated that, after Ensure infusion, feeding was similar between the groups (Figure 2E). Thus, despite baseline differences, intragastric nutrient infusion reduced feeding more strongly in regularly exercising mice.

Because intragastric nutrient delivery suppressed feeding more strongly in active mice, we next asked whether regular exercise alters brain responses to gut nutrient signals. We combined the intragastric infusion protocol with 18-fluorodeoxyglucose (FDG*)* positron emission tomography (PET), to determine brain-wide activity patterns (Figure 3A). In both regular wheel runners and sedentary controls, intragastric nutrient delivery in anaesthetized mice after an overnight fast caused an activation of a substantial proportion of brain regions, as determined by increased ^18^FDG uptake (Figure 3B). Detailed analysis and statistical comparison of activity patterns between runners and controls revealed augmented activation in numerous brain regions in mice that were regularly running (Figure 3C–I, S2A, B). Several of these regions were previously found to include neuronal populations that regulate consumption, including the insular cortex (Ins [42], [43]) and (para-) subthalamic region (STN [44], [45]; Figure 3D,E, H). In addition, we observed increased activation in regular runners in brain regions that are associated with motivational aspects of feeding behavior, including the lateral septum (LS [46], [47]), left dorsal hippocampus (dHip [48], [49]) and subiculum (Sub [50]), and additional brain regions (Figure 3D,F, G, I, S2A-B). These findings suggest that regular exercise is associated with increased brain-wide responses to gut nutrient delivery.

**Figure 3:**
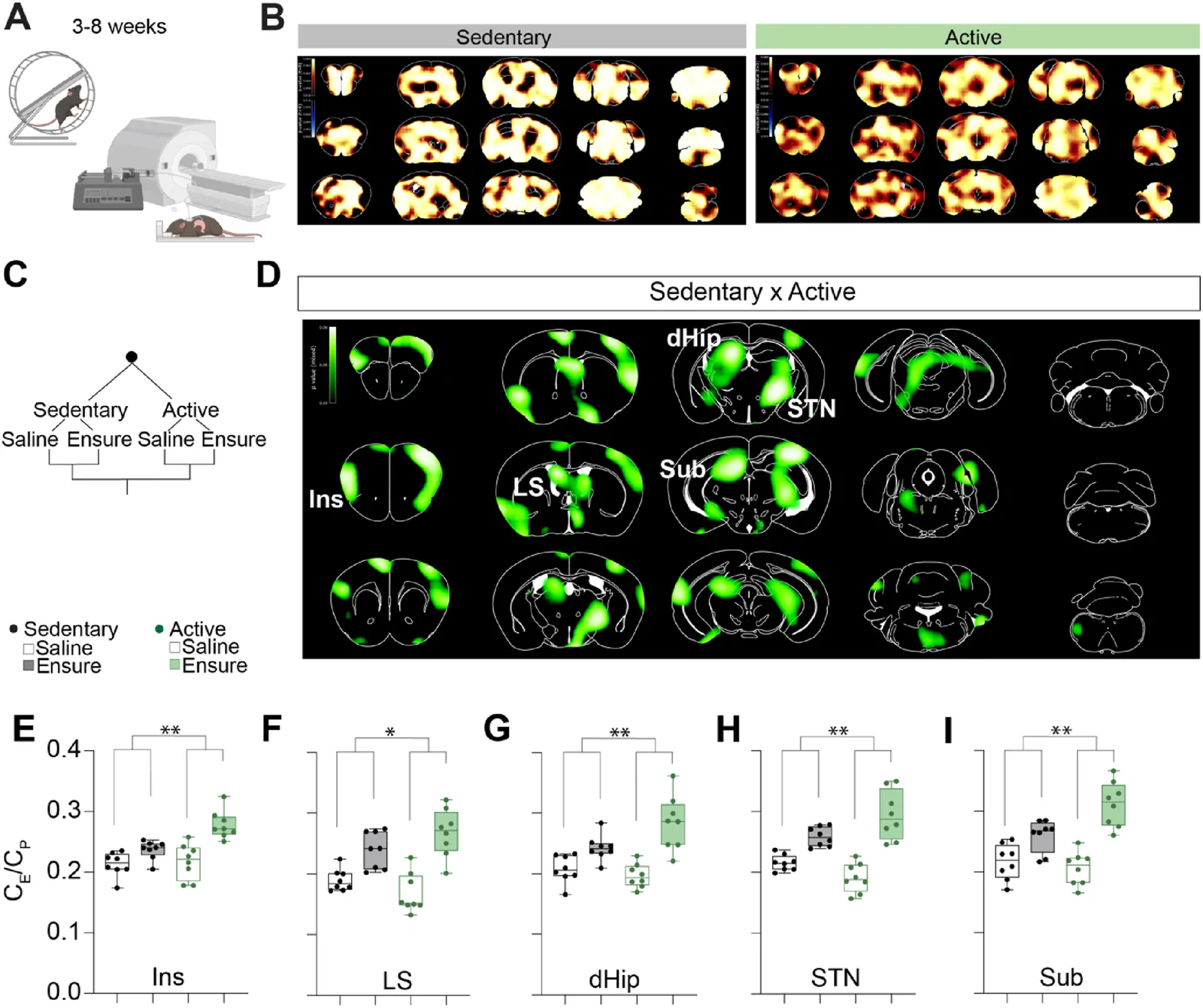
**Regular exercise augments brain responses to intragastric nutrient infusion.A,** Schematic showing experimental design. 3–8 weeks after initiation of a voluntary wheel running (active) or sham (sedentary) protocol, overnight fasted mice received an intragastric administration of Ensure at the beginning of ^18^FDG-PET measurements. **B,** Brain activation pattern after intragastric Ensure infusion in sedentary and active mice as assessed by ^18^FDG uptake (p values from voxelwise paired t-test (saline vs. Ensure) are indicated by color bar). n = 8 mice. **C,** Schematic for two-way analysis of brain activation patterns (^18^FDG uptake) between different experimental groups (sedentary vs. active) and conditions (saline vs. Ensure) with two-way ANOVA. **D,** Within group comparison (active vs. sedentary) of brain activation pattern after intragastric infusion of saline vs. Ensure as assessed by ^18^FDG uptake. p values from voxelwise two-way ANOVA are indicated by color. **E-I,** Statistical comparison of activation patterns in the left insula (Ins, ∗∗p = 0.0087, **E**), left lateral septum (LS, ∗p = 0.0294, **F**), left dorsal hippocampus (dHip, ∗∗p = 0.0053, **G**), right subthalamic region (STN, ∗∗p = 0.0015, **H**), and left subiculum (Sub, ∗∗p = 0.0038, **I.**) following Ensure infusion in sedentary and active mice. (two-way repeated measures ANOVA). n = 8 mice. Values are presented as mean ± SEM. Circles represent individual animals. Mice are from multiple litters.

Feeding reduction upon gut stimuli is mediated by various signaling mechanisms, including the release of peptides and hormones from enteroendocrine cells, such as cholecystokinin (CCK) [30], [51], [52], [53], [54]. A recent study found that regular wheel running increases the food intake-suppressing action of CCK in mice [38]. To test the contribution of CCK to the brain activation patterns following intragastric nutrient infusion in active mice, we performed ^18^FDG-PET measurements following i.p. injections of CCK-8 (2.5 μg/kg), or isovolumetric saline (Fig. S3C). We first focused our analysis on brain regions that showed significant differences in activity following intragastric infusion of Ensure by modelling the interaction of the experimental group (sedentary vs. active) and treatment (saline vs. CCK-8) for Ensure-sensitive peak voxels (Figure 3D–I). We did not find significant changes in the areas sensitive to Ensure with CCK-8 (Figure 3D–I, S2D). Instead, our brain-wide analysis demonstrated that CCK administration resulted in different activity responses in the dorsal striatum (DS), left substantia nigra (SN), right arcuate nucleus of the hypothalamus (ARC), right periaqueductal grey (PAG), left STN, and right lateral reticular nucleus (lRt, Figure 2F–L).

We next tested whether CCK contributes to the feeding reduction that we observed in runners following intragastric nutrient delivery (Fig. S3A, B). When CCK-8 was administered following an overnight fast, post-fast refeeding was not altered in both groups (Fig. S3C). We next probed the feeding effects of CCK-8 administration at the beginning of the dark cycle (Fig. S3D). We observed a slight, yet not significant, decrease in feeding in active but not in sedentary mice (Fig. S3E). Together, these findings suggest that although exercise alters brain responses to CCK, enhanced sensitivity to this gut-derived satiation signal does not account for the broad neural and behavioral effects observed following intragastric nutrient delivery.

### 2.3 Regular exercise increases the gut nutrient-induced activation of distinct brainstem neurons

We next sought to identify neuronal populations that show increased responses in exercising mice. The area postrema and nucleus of the solitary tract (AP/NTS) are brainstem regions that act as relay station of gut-derived signals, and contain various neuron populations that regulate visceral feedback control of feeding behavior [24], [55], [56]. To determine altered activity of AP/NTS neurons in exercising mice, we analyzed changes in our PET data with peak voxels placed in these regions. While we found that both areas showed increased activity following intragastric Ensure delivery, we did not observe differences between active and sedentary mice (Figure 4A,B).

**Figure 4:**
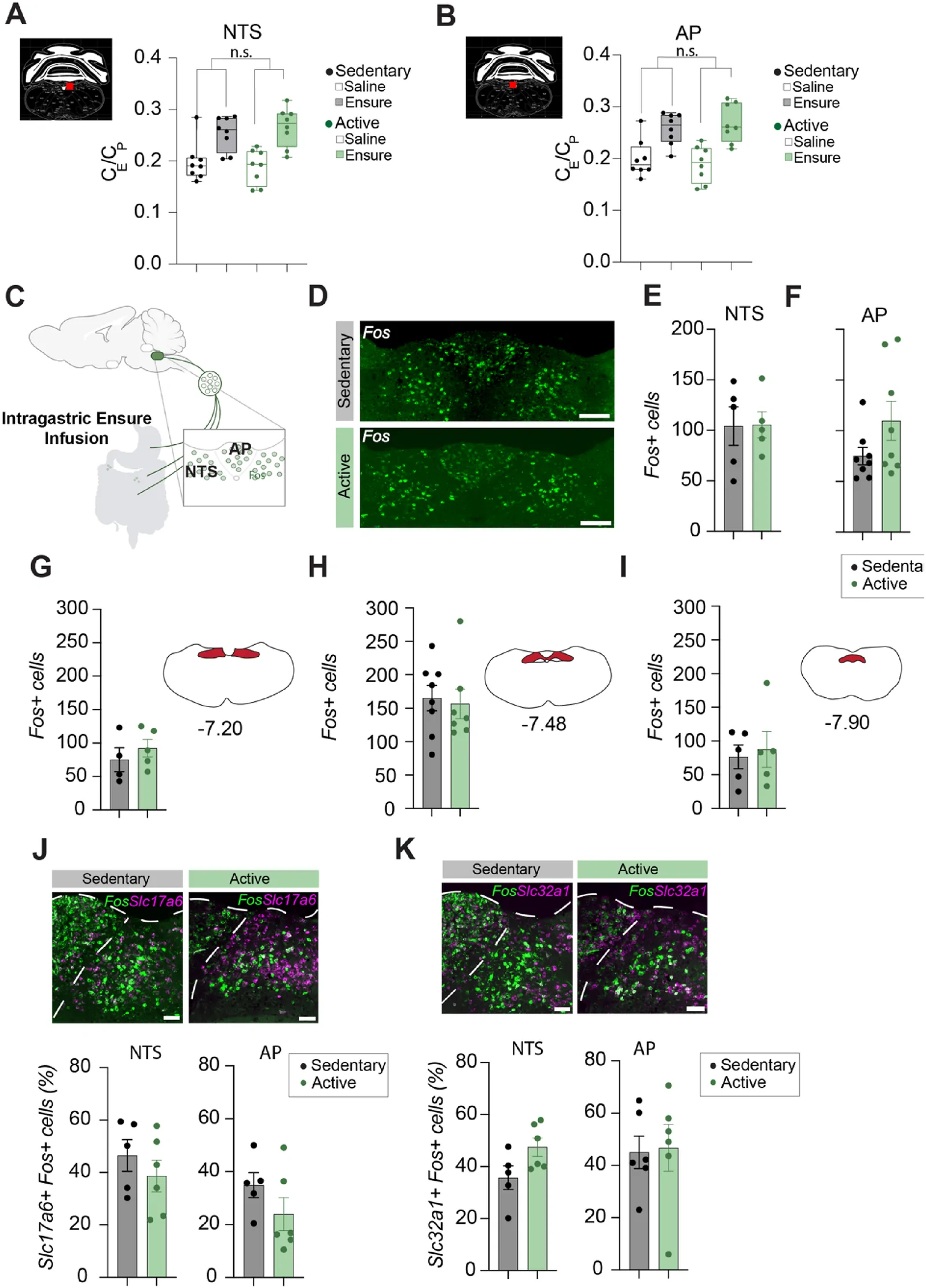
**Activation of dorsal brainstem neurons following intragastric nutrient delivery.A, B,** Analysis of activation in the NTS **(A)** and AP **(B)** in sedentary and active mice with PET. **C,** Schematic of the experimental design. After intragastric Ensure infusion, activity in the dorsal brainstem is assessed by *Fos* expression with FISH. **D-F,** Representative images **(D)** and analysis of *Fos* expression in the NTS (**E**) and AP (**F**) in sedentary and active mice. Scale bar = 200 μm. **G-I,** Analysis of *Fos* expression in the cranial (Bregma −7.20, **G**), medial (Bregma −7.48, **H**) and caudal (Bregma −7.90, **I**) division of the NTS as determined with FISH. **J,** Representative images and analysis of co-expression of *Fos* and Vglut2 (*Slc17a6*) in the NTS and AP as determined with FISH. **K,** Representative images and analysis of co-expression of *Fos* and Vgat (*Slc32a1*) in the NTS and AP as determined with FISH. Scale bar = 100 μm. Values are presented as mean ± SEM. Circles represent individual animals. Mice are from multiple litters. n.s. = not significant.

Because of the low spatial resolution of PET (0.4 mm × 0.4 mm x 0.8 mm voxels), we considered the possibility that activity differences in distinct neuronal populations in the AP/NTS region may have been missed by this approach. We therefore assessed alterations in activity patterns on a cellular level by examining expression of the neural activity marker Fos using fluorescent in-situ hybridization (FISH; Figure 4C). We found that the total number of *Fos*-expressing cells in animals sacrificed after intragastric Ensure delivery was not significantly different between regular wheel running and control mice (Figure 4D–F) when assessed for the entire NTS, or along the rostro–caudal axis (Figure 4G–I). Because neurons in the AP/NTS region can be broadly divided into excitatory and inhibitory [57], we additionally assessed co-expression of *Fos* and Vglut2 (*Slc17a6*), and *Fos* and Vgat (*Slc32a1*; Figure 4 J, K). We did not observe differences in active and sedentary mice along the rostro–caudal axis in *Fos* expression when using these markers for excitatory and inhibitory neurons. Thus, on an overall activity level, exercise does not alter neuronal responses upon intragastric nutrient delivery.

We next considered the possibility that regular exercise induces a selective shift in the activation of distinct populations of neurons in the AP/NTS. This possibility is of interest, because not only is the AP/NTS a highly heterogenous brain region that harbors more than 20 molecular distinct neuronal populations [57], but also because previous work demonstrated that some of these neurons reduce food intake [37], [56], [58], [59], [60] while others promote feeding [61], [62] when activated, and this depends on their molecular expression profile and/or anatomical location. To begin to explore this possibility, we performed single nucleus RNA sequencing (snRNAseq) of individual cells following intragastric infusion of Ensure or isovolumetric saline (Figure 5A). These snRNASeq studies were performed in a separate cohort of mice that were not subjected to exercise. We mapped the snRNAseq data onto previously published dataset [57] (Figure 5B), allowing an examination of the cellular composition of the captured region. Consistent with previous studies [57], [63], we found that the AP/NTS is highly heterogenous, containing numerous molecular distinct neuronal populations (Figure 5B). In addition, we found that sequenced cells did not cluster based on intervention (Fig. S5A) and include previously described excitatory and inhibitory neurons (Fig. S5B).

**Figure 5:**
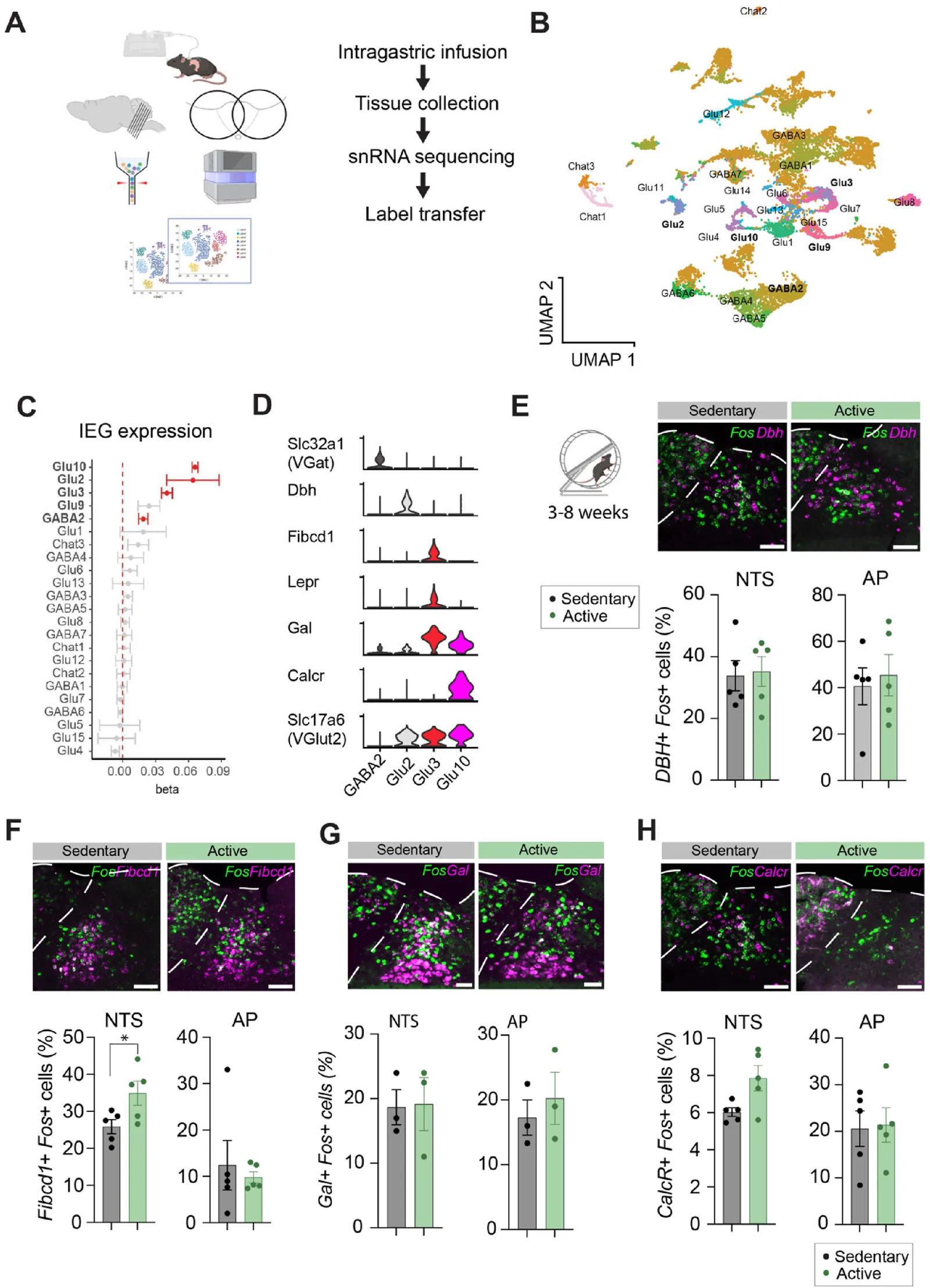
**Elevated FIBCD1 neuron activation after intragastric nutrient delivery in the NTS of physically active mice.A,** Schematic of the experimental design. Following intragastric infusion of Ensure, brains were collected, sectioned, and single nuclei were isolated from the dorsal brainstem containing the AP/NTS region to perform single-nucleus RNA sequencing (snRNAseq). n = 10 mice. **B,** UMAP of 23 clusters after label transfer from an existing DVC atlas [57]. **C,** Analysis of immediate early gene expression (IEG) revealed distinct neuron subtypes that are activated by intragastric infusion of Ensure (n = 5 mice) compared to saline (n = 5 mice). **D,** Violin plot of marker gene expression for clusters with significantly increased IEG expression (as determined in **C**). **E-H,** Histological assessment and analysis (bottom) of marker genes expression identified by snRNAseq (*Dbh*, *Fibcd1, Gal and CalcR*) and expression of *Fos* in brain sections from active and sedentary mice sacrificed after intragastric Ensure infusion. In the NTS of active mice significantly more Fos positive neurons co-express *Fibcd1* (**F**). ∗p = 0.0417 (unpaired two-sided t-test, n = 5 mice). Values are presented as mean ± SEM. Circles represent individual animals. Mice are from multiple litters. Scale bar = 100 μm.

Next, we examined the expression level of multiple immediate early genes (IEGs) in addition to *Fos* in the snRNAseq dataset (Figure 5C), reasoning that the aggregated response of multiple IEGs would allow to better define neuronal populations activated by intragastric Ensure delivery. This analysis revealed that four molecularly distinct neuron types showed increased neuronal activity: One GABAergic population (GABA2), and three glutamatergic populations (Glu2, Glu3, and Glu10; Figure 5C,D). Because excitatory AP/NTS neuron types have been shown to reduce food intake when activated [24], [27], [64], [65], we further characterized the glutamatergic neuronal populations, and determined genes that were enriched in these types. In the glutamatergic cluster Glu2, we observed enrichment of dopamine β-hydroxylase (*Dbh)* expression (Figure 5D, S4B), which marks a population of AP/NTS neurons that reduces feeding when activated [58]. In the Glu3 cluster, we observed expression of previously described genes in the AP/NTS regions [57], [66], including the *leptin receptor* (*Lepr*), and expression of *galanin* (*Gal*; Figure 5D, S4B). Interestingly, this glutamatergic neuron population was selectively marked by the expression of a previously uncharacterized gene encoding the endocytic acetyl group-binding transmembrane receptor fibrinogen C domain containing 1 (*Fibcd1*; Figure 5D, S4B). Neurons in cluster Glu10 cells, expressed the calcitonin receptor (*Calcr*), which marks a food intake-suppressing AP/NTS neuron population [60] (Figure 5D, S4B). Thus, our data suggest that in addition to the previously characterized DBH and CALCR neuron populations, which underlie visceral feedback regulation of feeding [58], [60], FIBCD1 neurons in the AP/NTS region are activated following intragastric nutrient delivery.

We next determined whether neuronal activity in the three nutrient-activated glutamatergic AP/NTS neuron types is augmented by regular exercise. We analyzed expression of *Fos*, *Dbh*, *Fibcd1*, *Gal* and *Calcr* following intragastric Ensure application in wheel running and control mice (Figure 5E–H). In line with sequencing results, FISH analysis revealed high *Fos* expression in *Dbh-*, *Fibcd1-, Gal-, and Calcr*-expressing AP/NTS neurons following intragastric Ensure infusion (Figure 5E–H). In *Dbh, Gal* and *Calcr* neurons, we observed no significant differences in *Fos* expression between regular wheel running and control mice (Figure 5E–H). Interestingly, we found a slight increase in the fraction of *Fos*-expressing NTS neurons co-expressing *Fibcd1* in regular wheel running mice (Figure 5F). We did not observe a change in marker gene expression between groups (Fig. S4C-E). Furthermore, *Fibcd1*-and *Calcr*-expressing neurons showed only minimal overlap in the NTS and AP (Fig. S4F, G), indicating a synergistic activity of these cells following nutrient delivery into the gastrointestinal tract. Together, these findings indicate that regular physically activity selectively increases the responsiveness of FIBCD neurons in the NTS to intragastric nutrient delivery.

Finally, we assessed whether FIBCD1 is a molecular marker of brainstem neurons that is conserved in humans. We performed FISH on brainstem sections from human tissues containing the AP/NTS with probes targeting *FIBCD1* (Figure 6A,B). In addition, we analyzed *SLC17A6* (Vglut2), and *LEPR* expression in human sections because these two markers were enriched in *Fibcd1*-expressing AP/NTS neurons in mice (Figure 5D, S4B). We found that in the human brainstem *FIBCD1* marks an NTS/AP cell population that highly co-expresses Vglut2 and *LEPR* (Figure 6C–E). Thus, as in mice, FIBCD1 marks a distinct population of excitatory neurons in humans in the AP/NTS region.

**Figure 6:**
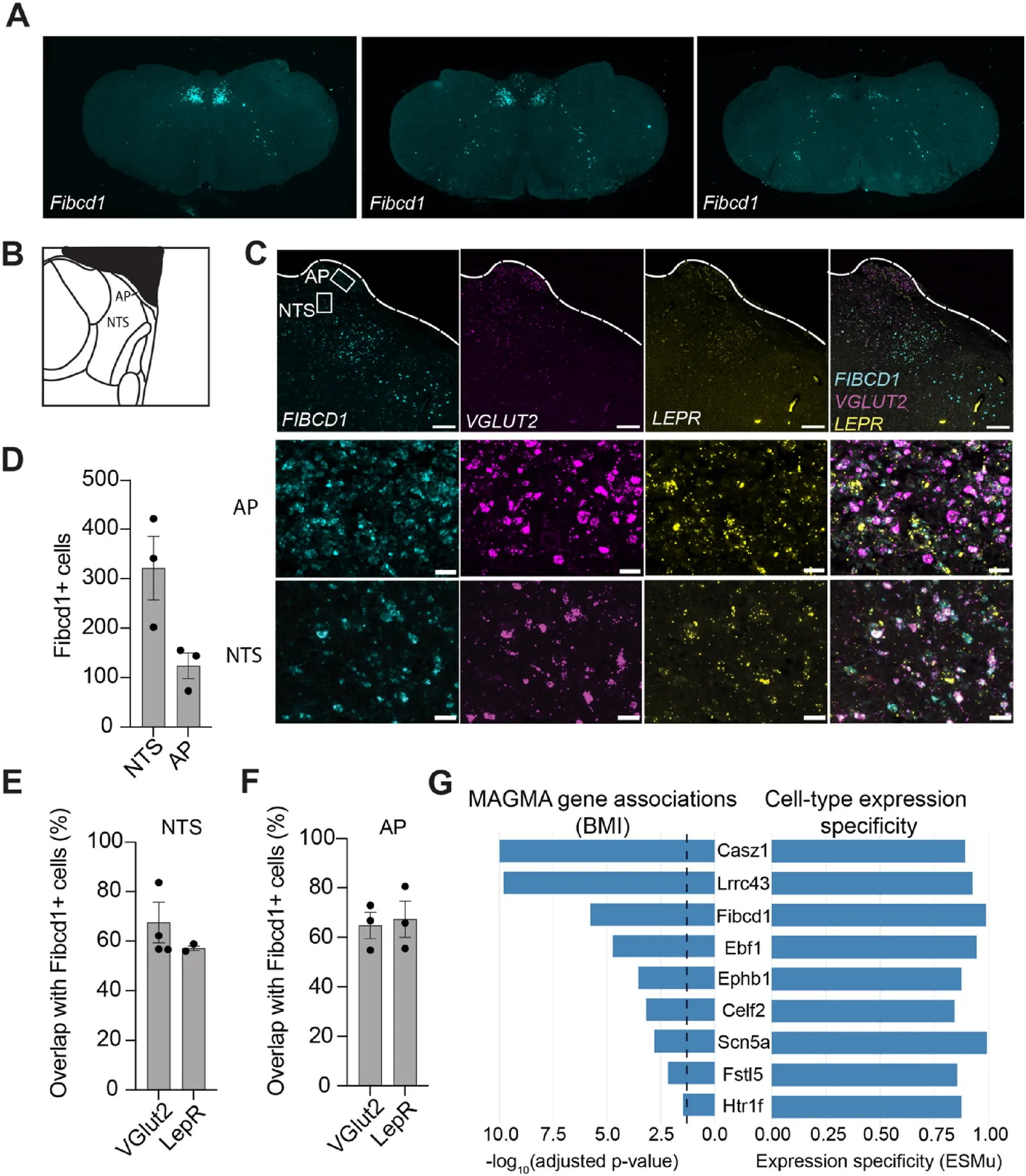
**FIBCD1 neurons in humans.A,***Fibrinogen c domain containing 1* (*Fibcd1*) expression in the mouse dorsal brainstem (rostral to caudal sections). **B,** Schematic overview of the human dorsal brainstem, according to the Allen Human Brain reference atlas. **C,** Representative images showing expression of *FIBCD1*, *VGLUT2* and leptin receptor (*LEPR*) in the NTS and AP in human brainstem sections, as determined by FISH. Scale bar = 500 μm (overview) or 50 μm (zoom in). **D,** Number of *FIBCD1*-expressing cells in the human NTS and AP. n = 3. **E, F,** Co-expression analysis of *FIBCD1*, *VGLUT2*, and *LEPR* in human NTS (**E**) and AP (**F**) sections. n = 3. **G,** Left: MAGMA gene-level association with BMI [67] shown as −log_10_(Bonferroni-adjusted *p*-value); dashed line indicates genome-wide significance (adj. *p* < 0.05). Right: CELLEX expression specificity (ESμ) scores for the same genes in the Glu3 hypothalamic neuronal cluster [57]. Shown are genes that are both significantly associated with BMI and specifically expressed in Glu3 (ESμ>0.8). Castor Zinc Finger 1 (Casz1), Leucine Rich Repeat Containing 43 (Lrrc43), EBF Transcription Facotr 1 (EBF1), EPH Receptor B1 (Ephb1), CUGBP Elav-Like Family Member 2 (Celf2), Sodium Voltage-Gated Channel Alpha Subunit 5 (Scn5a), Follistatin Like 5 (Fstl5), Hydroxytryptamin Receptor 1F (Htr1f). Gene names are given as mouse orthologs. Values are presented as mean ± SEM. Circles represent individual human samples.

To further determine the potential relevance of FIBCD1 neurons in energy balance regulation in humans, we used a previously published analysis [57], in which cell types were identified based on marker genes significantly enriched in body-mass-index (BMI) GWAS loci [67]. We found that the Glu3 population, which contains the *Fibcd1*-expressing neurons in mice, was among the cell types whose marker genes showed significant overrepresentation in BMI-associated loci (CELLECT, *p* = 2.6x10^−3^; Figure 6F). To identify individual genes driving this enrichment, we intersected MAGMA gene-level BMI associations (Bonferroni-adjusted *p* < 0.05) with CELLEX expression specificity scores for Glu3 (ESμ>0.8). This yielded ten genes that were both genome-wide significant for BMI and highly specific to Glu3, including *FIBCD1* (Fig. 6F). Thus, FIBCD1 neurons are potential candidates for future studies in body-weight regulation in humans, particularly in the context of regular exercise.

## 3 Discussion

One of the most widely recommended strategies for maintaining stable body weight is regular physical activity [1], [2], [68], [69]. Epidemiological and interventional studies consistently demonstrate that habitual exercise reduces the risk of obesity and metabolic disease, even in environments with access to calorie dense food [70]. In addition to increasing EE, regular exercise is associated with altered feeding behavior and improved long-term energy balance regulation [12], [71], [72], [73], [74]. However, the neurobiological mechanisms through which exercise modifies feeding control remain incompletely understood. In particular, it has remained unclear whether regular exercise alters gut-brain communication, a central pathway through which gastrointestinal nutrient signals regulate appetite and feeding behavior [17], [18], [22], [24], [25]. Addressing this question experimentally has been challenging because conscious food choices and learned feeding behaviors are difficult to disentangle from the physiological processing of visceral nutrient signals.

Here, we used intragastric nutrient infusion to directly probe gut-derived signaling in mice exposed to regular voluntary wheel running. This approach enabled us to isolate visceral nutrient sensing mechanisms from oral sensory cues and learned feeding behaviors associated with food consumption. We observed that, despite a marked increase in EE, particularly during the active phase, physically active mice did not proportionally increase caloric intake. Particularly following overnight fasting, a condition that further increased wheel running activity and EE, compensatory post-fast refeeding was not enhanced. Instead, intragastric nutrient delivery suppressed feeding more strongly in exercising mice than in sedentary controls. Importantly, this reduction exceeded the caloric value of the infused nutrients, indicating enhanced sensitivity to gut-derived anorexigenic signals rather than simple caloric compensation. Importantly, mice showed minimal wheel running during the post-fast refeeding period, arguing against the possibility that reduced feeding reflected competition between exercise and food seeking behavior [75].

Our PET imaging experiments demonstrated that intragastric nutrient delivery robustly activated distributed brain regions associated with feeding regulation, motivational processing, and visceral sensory integration. Multiple regions previously implicated in appetite control, including the dHip, LS, and STN [44], [45], [46], [47], [48], [49], [76], exhibited enhanced activation in mice subjected to regular exercise. The broad nature of these responses suggests that regular physical activity induces adaptations that increase the gain of gut-brain signaling across interconnected neural networks. Because the observed activity patterns accumulated over the recording period, they likely reflect integrated processing of nutrient-related signals during ongoing digestion and absorption, which involve coordinated signaling from multiple gastrointestinal organs over extended time scales [18], [24], [25], [77].

However, our findings do not exclude the possibility that exercise-induced adaptations in other visceral and autonomic signaling pathways contributed to the enhanced brain responses observed following intragastric nutrient administration. Regular physical activity induces substantial adaptations in cardiovascular and respiratory function, which are accompanied by changes in autonomic and interoceptive signaling to the brain [78], [79]. Signals arising from the heart, lungs, vasculature, and other visceral organs converge on the NTS and other regions of the central autonomic network and could therefore contribute to the responses detected by PET [80], [81], [82]. Similarly, exercise alters gastrointestinal physiology beyond nutrient sensing, including gastric distension and gastrointestinal motility, which can engage neural pathways independently of the nutrient content of the gut [83]. For example, parasubthalamic neurons respond to gastric distension independently of nutrient content and participate in the regulation of feeding [84]. Thus, the enhanced responses observed following intragastric nutrient delivery in exercising mice may reflect altered integration of nutrient-related and other visceral signals rather than an exclusive increase in nutrient sensitivity. Nevertheless, we found that FIBCD1 neurons were among the most strongly activated neuronal populations in the NTS following intragastric nutrient delivery in sedentary mice, supporting a preferential association of this population with gut-derived signals associated with nutrient ingestion. This is consistent with recent findings showing that FIBCD1 neurons are activated by a long-acting neurokinin 2 receptor (NK2R) agonist that suppresses food intake and body weight while increasing EE [66]. Importantly, our PET approach also does not resolve all neural adaptations that may contribute to exercise-induced changes in feeding. In particular, [18F]FDG uptake provides a measure of glucose metabolism but cannot distinguish the activity of specific neurotransmitter systems. Monoaminergic pathways, including dopaminergic circuits, are strongly implicated in the regulation of motivation, reward, feeding, and gut-brain signaling and are themselves modulated by physical activity [85], [86]. Thus, additional adaptations in monoaminergic signaling may contribute to the altered behavioral responses observed in exercising mice but cannot be directly assessed with the present approach. Future studies combining cell type-specific recordings and circuit manipulation with measurements of visceral and monoaminergic signaling will be required to define how exercise reshapes the integration of nutrient and other internal state signals across the brain.

The dorsal vagal complex of the brainstem (DVC), including the AP and NTS, represents a major entry site for visceral nutrient related signals into the brain [19], [23], [24], [27], [87], [88]. Although overall activity within this region was not altered by exercise at the level detectable by PET or total Fos expression, activity mapping on a cellular level revealed selective recruitment of molecularly distinct neuronal populations following intragastric nutrient delivery. Among these were *Dbh*- and *Calcr*-expressing neurons, both previously implicated in appetite suppression and visceral feedback regulation [58], [60].

FIBCD1 neurons were anatomically and molecularly distinct from previously characterized feeding regulatory populations in the DVC, including glucagon-like peptide-1 receptor-expressing neurons [57], in addition to DBH and CALCR. Importantly, regular exercise selectively increased the nutrient-evoked activation of FIBCD1 neurons in the NTS, identifying this population as a candidate neural substrate through which exercise may alter gut-brain communication. However, the functions of FIBCD1 neurons and their causal involvement in the regulation of feeding behavior in the context of exercise remain to be elucidated. Nevertheless, several observations support their potential relevance to the regulation of food intake, body weight, and whole-body metabolism. Specifically, alongside the recent observation that FIBCD1 neurons are activated following NK2R agonist administration [66], our findings show that FIBCD1 neurons are conserved in the human dorsal brainstem and that genes enriched within this neuronal population significantly overlap with BMI-associated loci. Future studies employing selective manipulation, recording, and circuit-mapping approaches will be necessary to determine the activity dynamics and functions of FIBCD1 neurons and whether increased responsiveness of this brainstem neuronal population to gut-derived nutrient stimuli contributes to exercise-induced alterations in gut-brain communication and feeding control.

Previous studies have shown that regular exercise induces substantial adaptations in gastrointestinal physiology, including altered gut microbiota composition, remodeling of intestinal anatomy, and enhanced secretion of enteroendocrine hormones in response to luminal nutrients [11], [38], [89], [90], [91], [92], [93], [94], [95]. These adaptations are likely to influence gut-brain signaling pathways. In particular, enhanced sensitivity to the satiation signal CCK has been proposed to contribute to exercise-induced suppression of feeding in rodents and humans [7], [38], [94], [96]. Although we observed altered brain responses to peripheral CCK administration in some regions, it did not account for the broad enhancement of nutrient-evoked brain activity observed following intragastric nutrient infusion. Further, CCK-induced feeding suppression was modest. These findings suggest that increased sensitivity to CCK alone is unlikely to represent the primary mechanism underlying exercise the herein described augmentation of gut-brain communication. Instead, the observed adaptations may emerge from the coordinated interaction of multiple peripheral and central signals, including microbiota-derived metabolites, lipid signaling molecules, vagal sensory pathways, and muscle-derived factors such as myokines [11], [38], [89], [91], [92], [93], [95], [96]. Elucidating how these signals converge onto identified brainstem populations, including FIBCD1 neurons, will be an important direction for future work.

A key observation of our study is that regular wheel running promoted a sustained negative energy balance characterized by reduced adiposity without robust compensatory hyperphagia. Because mice consumed most calories during the dark phase, when wheel running activity was highest, enhanced gut-brain signaling may have promoted earlier meal termination and more efficient integration of nutrient feedback during feeding. Such adaptations could improve the precision with which ongoing nutrient intake is translated into behavioral regulation of appetite. In this context, our findings provide a physiological framework for previous observations in humans showing improved appetite control and altered responses to caloric preloads in habitual exercisers [15], [16], [97].

Overall, our findings demonstrate that regular exercise sensitizes gut-brain communication and enhances visceral nutrient signaling to the brain. Moreover, we find FIBCD1 neurons in the brainstem as a previously unrecognized neuronal population whose nutrient responsiveness is selectively augmented by habitual physical activity. These findings provide a neurobiological framework through which exercise may improve appetite regulation and energy balance. Understanding how exercise remodels gut-brain signaling pathways may help guide the development of therapeutic strategies that mimic beneficial aspects of physical activity for the treatment of obesity and metabolic disease.

## 4 Materials and methods

### 4.1 Animals

Mice were single housed at 22–24 °C in a 12 h light/12 h dark cycle with ad libitum access to water and standard rodent chow diet (ssniff, V1554/SAFE D30), unless food was withdrawn for a specific experiment. At the age of 8–10 weeks mice were assigned to either experimental group (active or sedentary), considering equal body-weight distribution. Home cages were equipped with a running wheel with unlimited access, or a sham wheel for the sedentary control group. Mice with insufficient running activity (less than 2 km per night) were excluded from analysis. All experimental procedures were conducted in compliance with protocols approved by the local authorities (Bezirksregierung Köln and Dyreforsøgstilsynet Denmark).

### 4.2 Metabolic phenotyping

Measurements of food intake, running activity, EE, respiratory exchange ratio and locomotion was performed using an automated indirect calorimetry system (TSE phenomaster). Mice remained in their home cages for the duration of the measurement. For fasting-refeeding experiments, food was removed 1 h prior to the beginning of the dark cycle and reintroduced 16 h later. Fat and lean mass were determined using a magnetic resonance scanner.

### 4.3 Food intake measurements

Mice were handled daily for 7 consecutive days before food intake measurements. At the day of the experiment, food was removed 1 h before the start of the dark cycle. Intragastric infusion of a mixed meal solution (Ensure, 0.5 mL, 1 kcal/mL, 6 mL/h flow rate) was performed at the beginning of the dark cycle. Food was reintroduced 25 min later, and cumulative food intake was measured for 2 h. For refeeding experiments, food was removed, and mice were equipped with a fresh cage 1 h before the beginning of the dark cycle of the previous day. Mice received an intragastric nutrient infusion (Ensure, 0.5 mL, 6 mL/h flow rate) after 16 h of fasting. Food was reintroduced 25 min later, and cumulative food intake was manually monitored for 2 h. All measurements were performed in two consecutive weeks in a randomized crossover design. Control groups were attached to the infusion system without intragastric infusion.

### 4.4 Statistical analysis

#### 4.4.1 Experimental design and randomization

Mice were assigned to either the sedentary or active experimental group at 8–10 weeks of age using age- and body-weight-matched littermates, with balanced body-weight distribution between groups. Active mice had unrestricted access to a functional running wheel, whereas sedentary control mice were housed with a non-movable sham wheel.

For experiments involving intragastric nutrient delivery or CCK administration, measurements were performed using a randomized crossover design over two consecutive weeks.

#### 4.4.2 Sample size determination

No formal a priori sample size calculation was performed. Sample sizes were selected based on prior experience with comparable voluntary wheel-running, metabolic phenotyping, intragastric infusion, PET imaging, and histological experiments and included anticipated technical losses. The number of independent animals is indicated in the corresponding figure legends.

#### 4.4.3 Exclusion criteria and missing data handling

Mice underwent voluntary wheel running for 3–4 weeks before the start of experimental procedures. 24-hour running performance was assessed, and mice achieving a total running distance of less than 2 km/day, averaged over multiple days during the week preceding the experiments, were excluded from analysis according to the prespecified exclusion criterion.

For PhenoMaster data, whole datasets from individual mice were excluded if device failure resulted in incomplete data for a given experimental procedure to preserve the relationship between related measures (e.g. food intake and EE). Ad libitum feeding and fasting-refeeding experiments were evaluated separately.

No animals were excluded from the snRNAseq experiment. Processing and statistical analysis of the snRNAseq data were fully scripted and applied identically to all samples. Nucleus-level quality-control criteria are described in section 4.10.4.1.

#### 4.4.4 Outcome measures and statistical models

Individual mice were considered the experimental unit for all analyses. For snRNAseq, individual nuclei were used for cell-level analyses, whereas individual animals were considered the biological replicates for group-level comparisons. For histological analyses, measurements obtained from multiple brain sections or technical replicates within an animal were averaged before statistical testing. Data are presented as mean ± standard error of the mean (SEM), and individual data points represent individual animals unless otherwise indicated. For snRNAseq, individual data points in cell-level visualizations represent individual nuclei.

Body-weight, body-composition, histological measurements, and other outcomes obtained once per animal were compared between experimental groups using two-sided unpaired Student's t-tests.

PhenoMaster data, including food intake, EE, RER, and wheel-running activity, were continuously measured for each mouse. Data were aggregated into predefined light/dark or fasting/refeeding intervals, as appropriate. For EE, the area under the curve (AUC) was calculated over the respective analysis interval for each individual animal. For RER, mean values were calculated for each predefined phase, including the light and dark phases or fasting and refeeding periods, as appropriate, for each individual animal. Group differences were assessed using two-sided unpaired Student's t-tests.

Food intake measurements collected at multiple time points were analyzed using a mixed two-way ANOVA (treatment × time) with repeated measures for time. The group × time interaction was used to determine whether the temporal pattern of food intake differed between active and sedentary mice. Where significant, prespecified pairwise comparisons were performed using Sidak's post hoc test.

For PET imaging, statistical testing was performed for each image voxel by either application of a paired t-test between two conditions (Fig. 3B) or by application of a mixed two-way ANOVA to determine significant interactions between two factors (saline/CCK × sedentary/running or saline/Ensure × sedentary/running). In the mixed two-way ANOVA the factor sedentary/running was treated as independent (‘between’) and the factor saline/CCK or saline/Ensure as repeated measures (‘within’). Statistical calculations were performed using the Python module scipy.stats (function: ttest_rel) and the package pingouin (function mixed_anova). No multiple-test correction was applied. For presentation only, three-dimensional maps of p-values were recalculated on a 0.1 × 0.1 × 0.1 mm grid from the original dataset using trilinear interpolation.

#### 4.4.5 Blinding

For histological analyses, all data were evaluated by a single experimenter who was blinded to the experimental group. Blinding was not performed in any other experiment.

### 4.5 Positron emission tomography (PET)

Positron emission tomography (PET) imaging was performed using an Inveon preclinical PET/CT system (Siemens). CCK or vehicle injections were performed 30 min prior to t = 0 of the imaging protocol. Intragastric infusion of a mixed meal solution (Ensure, 1 mL, 1 kcal/mL) or an isovolumetric volume of 0.9% NaCl was initiated at t = 0 of the imaging protocol. Mice were anesthetized with 2% isoflurane in a 65%/35% nitrous oxide/oxygen gas mixture and positioned on a dedicated mouse carrier (MEDRES, Germany) carrying two mice. Body temperature was maintained at 37.0 ± 0.5 °C using a thermostatically controlled water-heating system.

For radiotracer administration, a catheter consisting of a 30G cannula connected to polyethylene tubing (inner diameter 0.28 mm) was inserted into the tail vein and secured with a drop of glue. Each mouse received 7–8 MBq of [18F]FDG in 50–100 μL saline via the tail vein after initiation of the PET scan. Emission data were acquired for 45 min. Thereafter, animals were automatically transferred to the CT gantry, and a CT scan was performed (180 projections over 360°, 200 ms, 80 kV, 500 mA). CT data were used for attenuation correction of the PET data, and the CT image of the skull was used for image co-registration.

Plasma glucose levels were determined from a tail vein blood sample using a standard glucometer (Bayer Contour Next, Bayer Vital GmbH) after removal of the tail vein catheters. PET data were histogrammed into time frames of 12 × 30 s, 3 × 60 s, 3 × 120 s, and 7 × 240 s, binned using full 3D binning, and reconstructed using the MAP-SP algorithm provided by the manufacturer. For co-registration, the imaging analysis software Vinci [98] was used. Images were co-registered to a three-dimensional mouse brain atlas constructed from the two-dimensional mouse brain atlas.

#### 4.5.1 Kinetic modeling

An image-derived input function was extracted from the PET data of the aorta, which could be identified in the first-time frame of each animal. Input function data were corrected for partial volume effects by assuming a standardized volume fraction of 0.6.

Parametric images of the [18F]FDG kinetic constants K1, k2, k3, and k4 were determined by voxel-wise fitting of the data to a two-tissue-compartment kinetic model (voxel size: 0.4 × 0.4 × 0.8 mm). K1 represents the rate constant for transport from blood to tissue, k2 for transport from tissue to blood, k3 for phosphorylation of [18F]FDG to [18F]FDG-6-phosphate, and k4 for dephosphorylation.

To detect variations in glucose metabolism, we used the ratio of tissue to blood glucose (CE/CP) rather than the direct metabolic rate. CE/CP was calculated from the rate constants as:

CE/CP=K1/(k2+k3/0.26)

CE/CP is a measure of glucose transport and, in contrast to the metabolic rate of glucose itself, is insensitive to changes in blood glucose levels. Because neuronal activation is accompanied by increased glucose transport, alterations in CE/CP can be used as a surrogate measure of changes in neuronal activation.

### 4.6 Gastric catheter implantation

Prior to surgery, mice received analgesics (Meloxicam and Buprenorphine). Mice were anesthetized with isoflurane (5% induction, 1.5–2% maintenance) in O_2_ (1 mL/min flow rate). Body temperature was maintained at 37 °C using a controlled heating pad. Eyes were protected from drying with 5% dexpanthenol creme (Bepanthen, Bayer, Germany) and the shaved incision sites were pre-treated with a local analgesic (Emla, Aspen, Germany). Two midline incisions were placed in the skin of the dorsal neck and abdomen. Skin incisions were connected by blunt dissection to build a subcutaneous tunnel. Next, the peritoneum was opened and the stomach was exposed using blunt forceps. To place the catheter (Instech Laboratories, Germany), the stomach wall of the corpus (glandular region) was punctured with jewelers-forceps. The catheter was inserted into the opening and secured into the stomach wall with a purse string using 6-0 silk. The peritoneum was closed using 4-0 silk. The catheter was guided through a small incision in the muscle of the right flank and anchored to the muscle tissue, and placed subcutaneously to exit through the neck incision. To ensure sterile access, the catheter was attached to a vascular access button (Instech Laboratories, Germany). Skin incisions were closed using 4-0 silk. Postoperatively, mice were given Metacam orally for at least 3 days, or longer until they reached their pre-operative body weight. Catheters were rinsed through the access button twice weekly, as well as after experimental procedures.

### 4.7 Tissue preparation (mouse)

Brain tissue was extracted after intragastric infusion of Ensure (Abbott Laboratories, USA) or isovolumetric saline (1 mL, flow rate 6 mL/h), following an overnight fast. For fluorescent in situ hybridization (FISH), mice received ketamine (100 mg/kg) and xylazine (18 mg/kg) 30 min after the end of the infusion, and were transcardially perfused with phosphate buffered saline (PBS), followed by 4% paraformaldehyde (PFA) in PBS. Brains were extracted and postfixed overnight in 4% PFA in PBS, and transferred to 20% sucrose in PBS. The brainstem was cut into 20 μm sections using a freezing microtome (Leica SM2010).

For snRNAseq, mice were deeply anesthetized with 5% isoflurane 30 min after the end of Ensure or saline infusion. Brains were extracted, immediately frozen in liquid nitrogen and stored at −80 °C. NTS tissue was collected using micropunches from 150 μm frozen sections prepared using a cryostat. Anatomical location was microscopically confirmed by collecting 10 μm sections every 300 μm for histological DAPI staining.

### 4.8 FISH with RNA scope

#### 4.8.1 Mouse

FISH was performed using the RNAScope multiplex fluorescent V2 assay (Advanced Cell Diagnostics, Bio-techne Manual Assay Kit Cat #323100) according to the protocol provided by the manufacturer. Free floating 20 μm NTS slices were washed in cold Dulbeccos phosphate buffered saline (DPBS, Gibco) 3 times, and mounted onto Superfrost Gold Adhesion glass microscope slides (Thermo Scientific). Slides were incubated for 12–16 h at 60 °C, postfixed in 4% PFA at 4 °C, and dehydrated through 4 dehydration steps with 50%, 70%, 100% and 100% ethanol at room temperature. After air drying for 5 min hydrogen peroxide was applied for 7 min, followed by target retrieval at 95 °C for 7 min. Slides were washed in distilled water and incubated in pure ethanol for 3 min. After air drying, brains were incubated with protease plus for 20 min at 40 °C in a moisture chamber, followed by 2 washing steps with distilled water. Probes for *Fos* (Advanced Cell Diagnostics, Bio-techne, Cat #316921-C2, Cat #316921-C3*), Dbh* (Cat #407851*), Slc32a1* (Cat #319191-C3*), Slc17ab* (Cat #319171), *Calcr* (Cat #494071)*, Fibcd1* (Cat #524021), or Galanin (Cat #400961) were hybridized for 2 h at 40 °C, and labeled with fluorophores Opal 570, Opal 520 and Opal 640. Slides were washed in washing buffer twice in between each step. Slides were mounted with DAPI (4′,6-diamidino-2-phenylindole) vector shield and imaged with a Slide Scanner (Olympus Vs 200).

#### 4.8.2 Human

Tissue blocks covering the caudal medulla were obtained from the Edinburgh Brain Bank (n = 5, 2M+3F, age >50y) in collaboration with Professor Colin Smith, following UK and DK legal and ethical guidelines. FISH was performed on formalin fixed paraffin embedded human brain blocks covering the caudal medulla. The tissue blocks were sectioned at 5 μm and mounted onto Fisher SuperFrost Plus glass slides (Fisher Scientific). Multiplex FISH was performed using the RNAscope LS multiplex fluorescent reagent kit and RNAscope LS 4-Plex Ancillary Kit Multiplex Reagent Kit (Advanced Cell Diagnostics, Bio-Techne, Cat #322800+#322830) together with iFluor488/iFluor546/iFlour594 (1:500, AAT Bioquest/VWR, Cat #45020/45,025/45,035) and Opal690 Fluorophore Reagent pack detection (1:500, Cat #FP1497001KT, Akoya Biosciences) on a Leica BOND RX Fully Automated Research Stainer (Leica Biosystems) according to the manufacturer's instructions. The human tissue sections were pre-treated for 30 min with HIER at 95 °C in ER2 followed by 15 min protease treatment and then hybridized with human-specific probes to detect mRNA transcripts for *LEPR-alltv* (captures all LEPR isoforms, #410378), *SLC17A6* (#415678-C2) and *FIBCD1* (#813128-C4) (Advanced Cell Diagnostics, Bio-Techne). As positive and negative controls, ACD 4-plex Multiplex Positive and Negative Control Mixes (Cat #321808 and #321838, ACD/Biotechne) were used. Slides were counterstained with DAPI and then cover slipped with Prolong TM Gold Antifade mountant (ThermoFisher Scientific). Fluorescent slide scans were acquired with an Olympus VS200 slide scanner (Olympus) using a 20x (0.8 NA) air objective and 385/470 (DAPI); 488/520 (iFl488); 546/570 (iFl546); 594/620 (iFl594); 650/690 (Opal690) filter sets. For analysis, sections containing the AP were selected (n = 3).

### 4.9 Fos analysis

For analysis of the activation marker Fos, images were read into ImageJ [99], and corrected for brightness and contrast. Individual channels were saved for further analysis in GNU Image Manipulation Program (GIMP, Microsoft). Cells were marked as *Fos*-positive in case of 3 or more fluorescent signals inside the cytoplasm, and subsequently investigated for co-expression of one of the molecular markers *Vglut2, Vgat, Dbh, Galanin, Calcr* or *Fibcd1* for individual NTS sections. For all markers brain sections of Bregma −7.92, Bregma −7.48 and Bregma 7.20 were investigated. Co-localization is reported as percentage of Fos positive neurons that co-express the individual marker.

### 4.10 Single-nucleus RNA sequencing (snRNAseq)

#### 4.10.1 Nucleus isolation

Frozen dorsal vagal complex samples were processed individually in randomized batches of 4–8 samples. Frozen tissue was first lysed in Nuclei EZ lysis buffer (Sigma, #NUC101) and manually homogenized using a KIMBLE 2 mL tissue douncer with pestle B (Sigma, #D8938) to isolate nuclei. The homogenate was incubated on ice for 5 min and then filtered through 40 μm mini cell strainers (Pluriselect, #43–10040). Nuclei were pelleted by centrifugation (1,000×*g*, 5 min, 4 °C) and resuspended in a 1:1 mixture of EZ lysis buffer and 50% OptiPrep™ iodixanol (Sigma, #D1556), resulting in a 25% iodixanol solution. The suspension was then layered onto a 29% OptiPrep™ iodixanol solution to create a density gradient. The gradient was centrifuged to separate nuclei from cellular debris (14,000×*g*, 22 min, 4 °C). Following centrifugation, the supernatant was removed and the pellet was resuspended in nuclei buffer consisting of PBS supplemented with 1% BSA, 2 mM Mg^2+^, and 40 U/μL Protector RNase inhibitor (Roche, #3335399001), and incubated for 15 min. Nuclei were subsequently centrifuged (1,000×*g*, 10 min, 4 °C), and the final pellet was resuspended in 100 μL nuclei buffer for nuclei hashing.

#### 4.10.2 Nuclei hashing and sorting

A TotalSeq™ anti-Nuclear Pore Complex Proteins Hashtag antibody (0.5 μg/μL; BioLegend, A0451-A0465, #AB_2861054 to #AB_2861068) was added to each 100 μL nuclei suspension together with NeuN antibody (1:2,000; Millipore, MAB377X) and incubated for 30 min. Nuclei were washed by adding 1 mL nuclei buffer followed by centrifugation (1,000×*g*, 10 min, 4 °C) to minimize nuclei loss. Following removal of the supernatant, nuclei were resuspended in nuclei buffer containing 0.002 μg/μL 7-AAD.

Up to 28,000 single nuclei, representing all processed samples, positive for 7-AAD and NeuN were sorted using a SH800 cell sorter (Sony Biotechnology) equipped with a 70 μm sorting chip (Sony Biotechnology, #LE-C3207) directly into Chromium Next GEM Single Cell 3′ v3.1 reverse transcription buffer (10x Genomics). Samples were processed immediately for library preparation.

#### 4.10.3 Library preparation and RNA sequencing

snRNAseq libraries were generated using the Chromium Next GEM Single Cell 3′ kit (Dual Index v3.1; 10x Genomics). The 10x Genomics protocol was followed through the cDNA amplification step, at which point 1 μL of 0.2 μM HTO primers was added. cDNA amplification and SPRI cleanup were then performed according to the 10x Genomics protocol.

During the final SPRI cleanup, beads containing large cDNA fragments and the supernatant containing small amplicons, including HTO-derived cDNA, were separated into individual 0.2 mL tubes for library generation. To generate the RNA libraries, the 10x Genomics protocol was continued without modification using the bead-bound fraction. To generate the HTO libraries, 70 μL SPRI beads (Beckman Coulter, #B23317) were added to the isolated supernatant and incubated at room temperature for 5 min to bind HTO cDNA fragments. The beads were then washed twice on a magnetic rack with 200 μL 80% ethanol. Following the second wash, the tubes were briefly centrifuged and residual ethanol was removed. The beads were air-dried for 2 min and incubated in 40 μL EB buffer (Qiagen, #1014609) at room temperature for 2 min. The tubes were returned to the magnetic rack, and 39 μL of supernatant was transferred to a fresh tube.

To amplify and add sample indices to the HTO libraries, PCR was performed using 50 μL Kapa HiFi HotStart ReadyMix 2 × (Roche Diagnostics, #07958935001), 2.5 μL 10 μM SI PCR primer, 2.5 μL 10 μM Illumina TruSeq BioL_D70X_s primer, and 20 ng HTO cDNA. PCR was performed using the following cycling conditions: 2 min at 98 °C; 15 cycles of 20 s at 98 °C, 30 s at 64 °C, and 20 s at 72 °C; followed by 5 min at 72 °C and a 4 °C hold.

Following PCR, 120 μL SPRI beads were added to each sample and incubated for 5 min. The beads were washed twice on a magnetic rack with 200 μL 80% ethanol. Residual ethanol was removed, and the beads were air-dried for 2 min on the magnetic rack. Subsequently, 40.5 μL EB buffer was added to each sample, mixed by pipetting, and incubated for 2 min off the magnet. The tubes were returned to the magnetic rack, and 39 μL of supernatant was transferred to a fresh tube. DNA concentration was determined using a TapeStation High Sensitivity 1000 DNA kit (Agilent, #5067–5584) and a Qubit Fluorometer (Invitrogen, #Q32851).

#### 4.10.4 RNA sequencing

Single-nucleus libraries were sequenced on SP and S2 flow cells using an Illumina NovaSeq 6000 platform (Illumina), with Read 1, Index 1, and Read 2 lengths of 28, 8, and 94 bp, respectively. Libraries were sequenced to a depth of approximately 40,000 reads per nucleus for the endogenous RNA libraries and 10,000 reads per nucleus for the hashtag oligonucleotide (HTO) libraries.

##### 4.10.4.1 Data processing and analysis

###### 4.10.4.1.1 Sequencing and preprocessing

BCL files were demultiplexed into FASTQ files using bcl2fastq v2.19.01. RNA reads were aligned to an optimized mm10 transcriptomic reference GTF file using Cell Ranger v7.0 (10x Genomics), including intronic sequences. CellBender [100] was used to identify and remove contaminating ambient RNA.

Hashtag oligonucleotide (HTO) libraries were quantified using kb-count (Kallisto | Bustools v0.24.4 [101]) and subsequently processed using the Seurat package [102]. HTO counts were first center-log-ratio (CLR) normalized using the Normalize Data function. A Gaussian mixture model with two components was then fitted to each HTO distribution. Droplets positive for multiple HTOs were classified as doublets, whereas droplets negative for all HTOs were classified as negative. Within-sample doublets were additionally identified using the recover Doublets function from scDblFinder. All identified doublets were removed, and quality-control measures were calculated using AddPerCellQC() from Scuttle v1.4 (Bioconductor).

###### 4.10.4.1.2 snRNAseq data analysis: initial processing

Downstream analyses were performed using Seurat v4.3.0. For each 10x lane, nuclei classified as outliers based on UMI counts were removed. Raw RNA counts were then normalized using the NormalizeData function, and principal component analysis (PCA) was performed using the RunPCA function with the top 3,000 variable genes as input. Clustering was subsequently performed using the FindNeighbors and FindClusters functions with the top 30 principal components as input. Contaminating non-neuronal nuclei were identified and removed based on the expression of established marker genes using CellAnnotatoR (https://github.com/khodosevichlab/CellAnnotatoR).

All raw single-nucleus RNA-seq data have been deposited in the EMBL-EBI BioStudies database under accession number E-MTAB-17845.

###### 4.10.4.1.3 Label transfer from reference single-cell datasets

To annotate neuronal populations, labels were transferred from an existing DVC atlas [57]. First, 10,000 nuclei were sampled from the annotated neuronal reference dataset to reduce computational load. Cell-type annotations were transferred using the FindTransferAnchors function with the top 30 principal components, and predictions were generated using the TransferData function. Predicted cell types and associated prediction scores were added to the query object as metadata and used to annotate neuronal populations.

To assess neuronal activity, a module score was calculated for each nucleus using the AddModuleScore function based on a curated list of immediate early genes (IEGs), including Fos, Btg2, Nr4a3, Fosb, and Junb. IEG module scores were calculated for individual nuclei and then averaged across nuclei within each animal to obtain an animal-level score. Only clusters represented by at least 10 nuclei per animal and at least two animals per treatment group were retained for downstream analysis. For each cluster, we fitted a linear mixed-effects model to nucleus-level IEG scores, with treatment as a fixed effect and a random intercept for animal (IEG score ∼ treatment + (1 | animal). The animal-level random intercept accounts for the non-independence of nuclei from the same animal. Pairwise comparisons were performed using the emmeans package. No correction for the number of clusters tested was applied at this screening stage. Clusters with a nominally significant p < 0.05 were considered activated by intragastric nutrient delivery and prioritized for follow-up.

### 4.11 GWAS analysis

Gene-level associations with body mass index were computed using MAGMA [103] on BMI GWAS summary statistics from Loh et al., 2018 [67]. SNPs were mapped to protein-coding genes using the NCBI build 37 annotations, with LD estimated from the European 1000 Genomes Phase 3 reference panel (1000 Genomes Project Consortium, 2015). CELLEX-derived [104] cell-type expression specificity scores for the Glut 3 hypothalamic neuronal cluster were obtained from Ludwig et al., 2021 [57]. Because MAGMA uses human Entrez IDs while CELLEX uses mouse gene symbols, MAGMA results were converted to mouse orthologs using org.Hs.eg.db (Carlson, 2019; https://doi.org/10.18129/B9.bioc.org.Hs.eg.db) and the homologene R package (Mancarci, 2019; DOI: https://cran.r-project.org/web/packages/homologene/index.html). Genes lacking a one-to-one ortholog were excluded. Genes both significantly associated with BMI (Bonferroni-adjusted *p* < 0.05) and specifically expressed in Glut_3 (CELLEX ESμ score>0.8) were identified by intersecting the two datasets.

## CRediT authorship contribution statement

**Diba Borgmann:** Writing – original draft, Visualization, Methodology, Formal analysis, Conceptualization, Investigation, Funding acquisition, Project administration, Writing – review & editing. **Dylan M. Rausch:** Visualization, Methodology, Formal analysis, Writing – review & editing. **Sofia Lundh:** Visualization, Methodology, Writing – review & editing. **Shad Hassan:** Formal analysis, Visualization. **Cansu Tokgöz:** Investigation. **Annika Glatzel:** Investigation. **Leonie Cabot:** Investigation, Writing – review & editing. **Malou C. ten Voorde:** Investigation. **Cecilie Bæch-Laursen:** Investigation, Writing – review & editing. **Paula Sanchis Tortosa:** Investigation, Writing – review & editing. **Claus Brandt:** Methodology, Conceptualization, Supervision, Writing – review & editing. **Heiko Backes:** Methodology, Investigation, Data curation, Writing – review & editing, Visualization. **Tune H. Pers:** Supervision, Methodology, Funding acquisition, Conceptualization, Writing – review & editing. **Henning Fenselau:** Writing – original draft, Visualization, Supervision, Funding acquisition, Conceptualization, Writing – review & editing, Methodology, Resources.

## Ethics declaration

This study was conducted in accordance with the ARRIVE (Animal Research: Reporting of In Vivo Experiments) guidelines. This study was approved by the LAVE, Germany. (Approval No. Az. 81–02.04.2020.A389)

## Declaration of competing interest

The authors declare that they have no known competing financial interests or personal relationships that could have appeared to influence the work reported in this paper.

## Data Availability

Data will be made available on request.
