# Supplementary material for: Regular exercise augments gut-brain communication and visceral feedback control of appetite
Source: Mol Metab. 2026 Oct 28;115:102463. doi: 10.1016/j.molmet.2026.102463 (PMC13753598; doi:10.1016/j.molmet.2026.102463)
Supplement: Supplementary file 1 [file mmc1.docx]

**K Extended Data Figure 1**

**Extended Data Figure 1: Metabolic phenotyping of regular runners and sedentary controls**

**A,** Absolute fat mass is decreased in in active mice after 4 weeks of voluntary wheel running. **p=0.0044 (unpaired two-sided t-test). n=8 mice.

**B,** Lean mass is not changed in mice after 4 weeks of voluntary wheel running. n=8 mice.

**C,** With ad libitum food access, 24 hour EE is significantly increased in physically active mice. **p=0.0025 (unpaired two-sided t-test). n=8 mice

**D,** With ad libitum food access, respiratory exchange ratio (RER) is not altered in active mice. n=8 mice.

**E,** With ad libitum food access, mice with running wheels (active) show no food intake difference over 24 hours. n=8 mice.

**F,** Twenty-minute feeding bouts for sedentary and active mice during the first 2 hours of the dark cycle. p=0.4057 (two-way ANOVA). n=8 mice.

**G,** During fasting, dark cycle EE significantly increased in physically active mice. ***p=0.005 (unpaired two-sided t-test)

**H,** Body weights before fasting (baseline), after a 16 hour fast (fasted), and after 24 hours of refeeding (refed) in sedentary and active mice. n=4-5 mice.

**I,** In the absence of food, mice showed elevated running activity during the dark cycle. **p=0.0021 (unpaired two-sided t-test).

**J,** 20-minute feeding bouts for sedentary and active mice during the first 2 hours of refeeding following a 16 hour overnight fast. *p=0.0230 (two-way ANOVA, Tukey’s post hoc test). n=7-8 mice.

**K,** Correlation of fasting EE and refeeding food intake (first 2 hours). n=7-8 mice.

**L,** Body-weight compensation after 24 hours of refeeding following a 16-h fast, compared to pre-fast body weight.

**M,** Active mice show a decreased RER during the first hours of refeeding, indicating changes in fuel type selection. n=7-8 mice.

**N,** Average RER during light cycle refeeding. p=0.0694 (unpaired two-sided t-test). n=7-8 mice.

**O,** EE was correlated to running activity during the dark cycle of ad libitum conditions (left, p=0.0193, r=0.7915; Pearson correlation; n=8 mice) and during fasting (right, p=0.0364, r=0.7853; Pearson Correlation; n=7-8 mice).

Values are presented as mean ± SEM. Circles represent individual animals. Mice are from multiple litters.

**Extended Data Figure 2**

**Extended Data Figure 2: Analysis of brain activation pattern following intragastric infusion or i.p. CCK-8 injection.**

**A,** Within group comparison (active vs. sedentary) of brain activation pattern after intragastric infusion of Ensure vs. saline as assessed by ^18^FDG uptake. p values from voxel-wise repeated measures two-way ANOVA are indicated by color. n=8 mice.

**B,** Statistical analysis of activation following intragastric infusion of ensure vs. saline in sedentary vs. active mice (p-values from voxel-wise repeated measures two-way ANOVA, analysis as described in Fig. 3c).

**C,** Experimental design. After 3-8 weeks of exercise mice received a low dose (2.5 µg/kg) i.p. injection of CCK-8 or saline as a control, followed by ^18^FDG-PET measurements.

**D,** Analysis of ^18^FDG-PET following i.p. injection of 2.5 µg/kg CCK-8 or saline as a control in sedentary and active mice. Interaction effects (group and treatment, s. also Fig. S2E) were calculated for Voxel-Of-Interests (VOIs) from intragastric infusion experiments (Fig. 3D-I) using repeated measures two-way ANOVA. CCK injection did not result in activation of nutrient-sensitive brain regions. n=6-10 mice.

**E,** Schematic for voxelwise repeated measures two-way ANOVA analysis of brain activation patterns (^18^FDG uptake) between different experimental groups (sedentary vs. active) and conditions (saline vs. 2.5 µg/kg CCK-8 injections).

**F,** Brain activation pattern after CCK-8 injection in sedentary vs. active mice as assessed by ^18^FDG uptake. p values from voxelwise repeated measures two-way ANOVA (Sedentary vs. Active x Saline vs. Ensure) are indicated by color bar.

**G-L,** Statistical comparison (voxelwise repeated measures two-way ANOVA) of activation patterns in the right dorsal striatum (DS, **p=0.0094, **G**), arcuate nucleus (ARC, **p=0.0221, **H**), subthalamic region (STN, **p=0.0015, **I**), substantia nigra (SN, *p=0.0114, **J**), periaqueductal grey (PAG, **p=0.0042, **K**) and lateral reticular nucleus (lRt, *p=0.0238, **L**) following CCK-8 injection in sedentary and active mice. n=6-10 mice.

Values are presented as mean ± SEM. Circles represent individual animals. Mice are from multiple litters.

**Extended Data Figure 3**

**Extended Data Figure 3: Food intake after CCK-8 administration.**

**A,** Schematic: CCK relays gut-derived signals and regulates feeding behavior.

**B,** Schematic shows experimental design. Mice were subjected to a voluntary wheel running (active) or sham (sedentary) protocol. After 3-8 weeks of running, all animals received an i.p. injection of CCK or saline in a randomized crossover design in two consecutive weeks 30 minutes before refeeding following a 16-h overnight fast. Cumulative food intake was measured for 2 hours.

**C,** Cumulative food intake after 0.5, 1, and 2 hours of refeeding following a 16 hour overnight fast and 2.5 µg/kg CCK-8 injections, or control injections of saline, in sedentary and active mice. (two-way ANOVA, Sidaks post hoc test). n=7 mice.

**D,** Schematic shows experimental design. Mice were subjected to a voluntary wheel running (active) or Sham (sedentary) protocol. After 3-8 weeks of running, all animals received an i.p. injection of CCK or Saline in a randomized crossover design in two consecutive weeks 30 minutes before the onset of the dark cycle. Cumulative food intake was measured for 2 hours.

**E,** Cumulative food intake after 0.5, 1, and 2 hours of dark-cycle feeding following 2.5 µg/kg CCK-8 injections, or control injections of saline, in sedentary and active mice (two-way ANOVA, Sidaks post hoc test). n=9-10 mice.

Values are presented as mean ± SEM. Circles represent individual animals. Mice are from multiple litters.

**Extended Data Figure 4**

**Extended Data Figure 4: Gene expression analysis in dorsal brainstem neurons following intragastric nutrient delivery.**

**A,** UMAP of NTS clusters after label transfer, color-coded for intervention (intragastric saline vs. Ensure infusion). n= 10 mice.

**B,** Violin plot shows expression levels of marker genes for all clusters. *Fibcd1* is uniquely expressed in Glu3 neurons. n= 10 mice.

**C-E,** Statistical analysis of expression of *CalcR*, *Dbh* and *Fibcd1* in NTS and AP of sedentary and active mice as determined by FISH. n=5-6 mice.

**F, G,** Representative images (**F**) and statistical analysis (**G**) of co-expression of *Fibcd1* and *CalcR* in NTS and AP in sedentary and active mice. Scale bar = 100µm. n= 5-6 mice.

Values are presented as mean ± SEM. Circles represent individual animals (FISH, **C-E, G**) or neurons (snRNAseq, **A**). Mice are from multiple litters.
